# Comparative assessment of image quality for coronary CT angiography with iobitridol and two contrast agents with higher iodine concentrations: iopromide and iomeprol. A multicentre randomized double-blind trial

**DOI:** 10.1007/s00330-016-4437-9

**Published:** 2016-06-07

**Authors:** Stephan Achenbach, Jean-François Paul, François Laurent, Hans-Christoph Becker, Marco Rengo, Jerome Caudron, Sebastian Leschka, Olivier Vignaux, Gesine Knobloch, Giorgio Benea, Thomas Schlosser, Jordi Andreu, Beatriz Cabeza, Alexis Jacquier, Miguel Souto, Didier Revel, Salah Dine Qanadli, Filippo Cademartiri, on behalf of the X-ACT Study Group

**Affiliations:** 1 Department of Cardiology, Friedrich-Alexander-Universität Erlangen-Nürnberg, Ulmenweg 18, 91054 Erlangen, Germany; 2 Department of Radiology, Centre Chirurgical Marie Lannelongue, Le Plessis Robinson, France; 3 University of Bordeaux, Centre de Recherche Cardio-Thoracique de Bordeaux, U1045, F-33000 Bordeaux, France; 4 CHU de Bordeaux, Service d’Imagerie Thoracique et Cardiovasculaire, F-33600 Pessac, France; 5 Department of Clinical Radiology, University Hospital Grosshadern, Munich, Germany; 6 Department of Radiological Sciences, Oncology and Pathology, Sapienza – University of Rome, ICOT Hospital, Latina, Italy; 7 Department of Radiology, University Hospital of Rouen, Rouen, France; 8 Department of Radiology, Saint Gallen Hospital, Saint Gallen, Switzerland; 9 Department of Radiology, Cochin Hospital, Paris, France; 10 Department of Radiology, La Charité, Berlin, Germany; 11 Ospedale del Delta, Ferrara, Italy; 12 Elisabeth-Krankenhaus Hospital, Essen, Germany; 13 Hospital Vall d’Hebron, Barcelona, Spain; 14 Hospital Clinico San Carlos, Madrid, Spain; 15 Department of Radiology, La Timone Adult Hospital, Marseille, France; 16 Complejo Hospitalario Universitario, Santiago de Compostela, Spain; 17 Department of Radiology, Louis Pradel Hospital, Lyon, France; 18 Department of Radiology, University of Lausanne, Lausanne, Switzerland; 19 Department of Radiology, Giovanni XXIII Hospital, Monastier di Treviso, Italy

**Keywords:** Coronary computed tomography angiography, Image quality, Contrast media, Iodine concentration, Safety

## Abstract

**Objectives:**

To demonstrate non-inferiority of iobitridol 350 for coronary CT angiography (CTA) compared to higher iodine content contrast media regarding rate of patients evaluable for the presence of coronary artery stenoses.

**Methods:**

In this multicentre trial, 452 patients were randomized to receive iobitridol 350, iopromide 370 or iomeprol 400 and underwent coronary CTA using CT systems with 64-detector rows or more. Two core lab readers assessed 18 coronary segments per patient regarding image quality (score 0 = non diagnostic to 4 = excellent quality), vascular attenuation, signal and contrast to noise ratio (SNR, CNR). Patients were considered evaluable if no segment had a score of 0.

**Results:**

Per-patient, the rate of fully evaluable CT scans was 92.1, 95.4 and 94.6 % for iobitridol, iopromide and iomeprol, respectively. Non-inferiority of iobitridol over the best comparator was demonstrated with a 95 % CI of the difference of [-8.8 to 2.1], with a pre-specified non-inferiority margin of -10 %. Although average attenuation increased with higher iodine concentrations, average SNR and CNR did not differ between groups.

**Conclusions:**

With current CT technology, iobitridol 350 mg iodine/ml is not inferior to contrast media with higher iodine concentrations in terms of image quality for coronary stenosis assessment.

***Key Points*:**

• *Iodine concentration is an important parameter for image quality in coronary CTA.*

• *Contrast enhancement must be balanced against the amount of iodine injected.*

• *Iobitridol 350 is non-inferior compared to CM with higher iodine concentrations.*

• *Higher attenuation with higher iodine concentrations, but no SNR or CNR differences.*

## Introduction

Coronary computed tomography angiography (coronary CTA) has become widely accepted in clinical practice [1–3]. Technology progress has increased the robustness and diagnostic performance of coronary CTA, resulting in improved image quality and lower radiation exposure [4–9].

The protocols for administration of intravenous contrast media (CM) are of major importance in coronary CTA, usually performed to identify coronary artery stenoses but also calcified and non-calcified plaques [10–13]. The optimal intravascular attenuation for coronary CT angiography is under debate [14, 15]. Several publications suggested that adequate opacification of the vessel lumen for the simultaneous identification of both calcified and non-calcified plaques requires a careful contrast injection protocol that achieves a lumen opacification of at least 300 HU [15–17]. In principle, the Iodine Delivery Rate (IDR) should be the reference when using different compounds for intraluminal enhancement [18]. However, most centres do not use this approach in clinical practice.

Higher iodine concentration of the injected CM is associated with higher attenuation [16, 19]. However, increasing the total amount of iodine injected could raise safety issues for patients at risk such as contrast induced nephropathy [20–22]. Therefore, adequate contrast enhancement must be balanced against the amount of iodine injected. Several studies have addressed the level of attenuation with different CM [14, 23, 24], but few studies evaluated the impact of their concentration on image quality [25]. Furthermore, whether differences less than 50 mg/ml of iodine concentration could affect image quality remains to be clarified. The present study compared a CM with iodine concentration of 350 mg iodine/ml (iobitridol) to two CM with higher iodine concentrations (iopromide 370 mg/ml and iomeprol 400 mg/ml) for coronary CTA. The main objective of the study was to demonstrate the statistical non-inferiority of iobitridol 350 compared to the best of the two comparators in terms of image quality and interpretability as measured by the rate of patients with CT scans evaluable for the identification of coronary artery stenosis.

## Material and methods

### Study design and patient enrolment conditions

This study was a non-inferiority, multicentre, randomized, double-blind, clinical trial on three parallel groups. Patients were included in 23 centres from five countries between November 2010 and September 2012 and randomized on a 1:1:1 ratio to undergo clinically indicated coronary CTA after injection of iobitridol, iopromide or iomeprol. The study was approved by each local ethics committees and the National Health Authorities. Written informed consent was obtained from each participating patient.

Symptomatic adult patients with suspected coronary artery disease (CAD), and scheduled for coronary CT angiography were enrolled in this study. Patients could not be included if they had both a contraindication to β-blocker medications and a baseline heart rate above 65 beats per minute (bpm). Additional reasons for exclusion were the presence of arrhythmias or non-sinus rhythm, coronary artery bypass grafts or stents, artificial heart valves, moderate to severe aortic valve stenosis, hyperthyroidism, clinical instability, severe renal failure or previous injection of any CM within 48 hours prior to the study.

### Patient preparation

β-blockers were mandatory if heart rate was >65 bpm. The specific drug, dose and mode of administration were selected according to site routine practice. A minimum dose of 0.8 mg of sublingual nitroglycerine spray was mandatory immediately before the CT examination. Other pre-medication was permitted, given according to operator preference, and recorded.

### Injection of contrast media

For each patient, one of three CM was delivered intravenously: Iobitridol 350 mg iodine/ml (Xenetix®, Guerbet, Aulnay-sous-Bois, France), iopromide 370 mg/ml (Ultravist®, Bayer Healthcare, Berlin, Germany) and iomeprol 400 mg/ml (Iomeron®, Bracco, Milan, Italy). Delivered volume and delivery rate of CM was consistent for the three CM but varied according to patient body weight (BW): 60 ml injected at 4 ml/s for a BW <60 kg, 75 ml at 5 ml/s for a BW between 60 and 80 kg, 90 ml at 6 ml/s for a BW >80 kg. Therefore, the iodine-delivery rate was lowest for iobitridol. CM was warmed and injections were followed by a 100 % saline flush of 75 ml administered at the same rate as the CM.

### Scan protocol

All coronary CTAs were performed on systems with at least 64 detector rows (single or dual source). The tube voltage was adapted to the patient’s BW depending on the equipment used in the site: either 120 kVp for all patients or 120 kVp for BW ≥80 kg and 100 kVp for BW <80 kg. Each patient first received a non-contrast acquisition for the quantification of coronary calcium, followed by a high-resolution contrast-enhanced acquisition. Non-contrast scans were acquired according to local protocol, mandating the use of 120 kV tube voltage and 3 mm thickness of the reconstructed cross-sectional images.

Test bolus or bolus tracking [26, 27] as well as dose reduction techniques (e.g. mAs modulation, scan duration, tube current and pitch) were chosen according to site specific routine. Retrospective ECG-gating was always allowed, whereas prospectively ECG triggered data acquisition was only permitted for patients with HR < 65 bpm.

Dose length product (DLP) and volume CT dose index (CTDIvol) were recorded for the non-enhanced and for the contrast-enhanced coronary CTA acquisition.

### Calcium scoring

The calcium score [13] was calculated on-site. Patients were then classified using the following standard scale [28]: 1 = normal (no calcium); 2 = mildly elevated (Agatston Score 1 to 100); 3 = moderately elevated (Agatston Score 101 to 400); 4 = highly elevated (Agatston Score 401 to 1000); 5 = severely elevated (Agatston Score > 1000).

### Image reconstruction for coronary CTA

Cross-sectional image data sets were reconstructed using the thinnest possible slice thickness and standard reconstruction kernels (i.e. medium-smooth) without implementation of iterative reconstruction algorithms in order to reduce software-related attenuation variability. The choice of the best temporal window was left to the local operators. Original axial DICOM data were transferred for off-site reading purposes to a dedicated image core laboratory.

### Image evaluation for detection of stenosis

All CTA images were assessed by two independent, experienced readers with more than 10 years of experience in coronary CT angiography, both fully blinded to the patients´ clinical characteristics and the CM used.

The primary endpoint was the rate of patients with evaluable CT scans, i.e. CT scans that made it possible to identify or rule out coronary artery stenoses in all segments of the coronary tree with a reference diameter of 1.5 mm or more. Using a 5-point scale, off-site readers graded the quality of all 18 segments of the Society of Cardiovascular Computed Tomography (SCCT) coronary segmentation model [29] (4 = excellent quality, full confidence without any doubts concerning the presence/absence of luminal stenosis; 3 = good quality, confidence concerning the presence/absence of luminal stenosis; 2 = moderate quality, relative confidence, with minor doubts concerning the presence/absence of luminal stenosis; 1 = poor quality, some doubts concerning the presence/absence of stenosis; 0 = non diagnostic, with relevant doubts concerning the presence/absence of stenosis). A patient’s CT scan was considered as evaluable for identification of coronary artery stenosis if none of the 18 coronary segments had a score of 0 (except for segments graded 0 due to an occlusion located proximally, which did not make the patient non-evaluable).

As a secondary endpoint, a per-patient image quality score was computed off-site by averaging segment quality scores within patients.

### Attenuation, signal and noise measurements

Arterial attenuation, signal-to-noise ratio (SNR) and contrast-to-noise ratio (CNR) constituted secondary endpoints. Arterial vascular attenuation was measured off-site as follows: one region of interest (ROI) of 2 mm^2^ minimum size and located in the lumen of the LAD, the LCX, the RCA and the LM coronary artery; one ROI of 100 mm^2^ minimum size in the ascending aorta and one ROI of 50 mm^2^ minimum size in the left ventricle. Attenuation of LM, ascending aorta and left ventricle was measured on pre and post contrast images.

The noise was measured in the aorta (100 mm^2^ minimum size, located at the level of left main origin), air-filled cavities (50 mm^2^ in bronchia or trachea) and muscle (25 mm^2^, thoracic wall) in post-contrast images and was used to derive SNR and CNR values.

### Stenosis assessment and patient management

The presence of significant stenosis (>50 % of the lumen) in the 18 SCCT segments was reported by on-site radiologists using a 5-point scale (5 = certainly yes; 4 = probably yes; 3 = doubtful; 2 = probably no ; 1 = certainly no).

On-site radiologists recorded the recommended management from the following list: no action, medication, invasive coronary angiography and other.

### Coronary track rate

In order to determine whether image quality is sufficient for automated segmentation as often used for evaluation, commercially available software (“Comprehensive Cardiac Analysis” on IntelliSpace Portal, Philips) was used to automatically track LAD, LCX and RCA up to their distal segments and the number of segments tracked per patient constituted the ‘coronary track rate’, which was assessed by a third independent off-site radiologist, also with more than 10 years of experience in cardiac CT.

### Clinical safety

All adverse events (AEs), including cardiac events as evidenced by ECG (performed up to 10 min post-injection), were reported from the patient’s signature of the informed consent up to 30 min after the examination. The intensity of AEs was classified on a 3-point scale, based on interference with daily activities: mild (no interference), moderate (moderate interference) or severe (the subject is unable to work). The causal relationship of the AE to the CM injected was defined according to the French Method of Causality Assessment [30].

Patient comfort and pain were assessed on-site using a self-administered questionnaire (5-point scale ranging from 1 (worst situation) to 5 (best situation)) and a visual analogue scale (VAS), respectively.

### Statistical analysis

Assuming an expected proportion of 90 % of patients with coronary CTAs evaluable for CAD diagnosis with the three investigated contrast agents, 424 assessable patients (3x141) were needed to ensure with 80 % power and 5 % two-sided type-one error that the lower limit of the 95 % confidence interval (95 % CI) of the difference between iobitridol 350 and the best of the two comparators (iopromide 370 or iomeprol 400) is not greater than the clinical non-inferiority margin set at -10 %.

Three patient populations were defined: all-included-patients (AIP), full analysis set (FAS), and safety set.

AIP population included all patients enrolled in the study and having signed the informed consent. FAS included all patients who underwent the examination and had available assessments of the primary endpoint for iobitridol 350, iopromide 370 or iomeprol 400 examinations. The safety set included all patients who had received at least one injection of contrast agent, regardless of the quantity.

For comparisons between the three groups, two-sided tests were performed at a 5 % level of significance. In case of multiple comparisons, significance level of each test was adjusted to ensure a 5 % overall significance level. Accuracy of estimates was computed with 95 % CIs.

Student’s t-test and the F-test were used for quantitative variables, whereas the Chi-square test was used for qualitative variables.

Multiple regression models were performed to identify potential relationship between calcium scoring and stenosis assessment, image quality and territory.

## Results

### Study population

A total of 468 patients gave their consent and therefore were included (58 % male; aged 57.8 ± 12.4 years). Sixteen patients were excluded: five patients did not have a CM injection, and in 11 patients off-site image assessment was not possible due to technical failures. Therefore, 452 patients were analysed in the FAS and 463 in the safety set.

There were no significant differences between the three groups in terms of demographics, clinical symptoms, risk factors and pre-CTA heart rate. No differences were noted in terms of requirement for β-blockers for the CTA procedure, calcium score and radiation dose (Table 1).

**Table 1:** Patients and procedure characteristics

Parameters	Iobitridol 350	Iopromide 370	Iomeprol 400	Total	Test
Demographics	N = 155	N = 160	N = 153	N = 468	
Age (years)	57.9 ± 12.2	58.7 ± 11.6	56.9 ± 13.4	57.8 ± 12.4	p = 0.457 (F)
Male gender	90 (58.1 %)	92 (57.5 %)	88 (57.5 %)	270 (57.7 %)	p = 0.993 (C)
Height (cm)	170.4 ± 10.5	168.9 ± 8.9	169.8 ± 10.0	169.7 ± 9.8	p = 0.380 (F)
Body weight (kg)	78.7 ± 15.4	76.6 ± 16.9	77.3 ± 14.0	77.5 ± 15.5	p = 0.445 (F)
Body mass index (kg/m^2^)	27.0 ± 4.3	26.7 ± 4.7	26.7 ± 3.6	26.8 ± 4.2	p = 0.736 (F)
CV risk factors					
Hypertension	79 (51.0 %)	84 (52.5 %)	82 (53.6 %)	245 (52.4 %)	p = 0.898 (C)
Diabetes	15 (9.7 %)	17 (10.6 %)	17 (11.1 %)	49 (10.5 %)	p = 0.916 (C)
Smoking	49 (31.6 %)	63 (39.4 %)	53 (34.6 %)	165 (35.3 %)	p = 0.347 (C)
Family history of CAD	58 (37.4 %)	62 (38.8 %)	67 (43.8 %)	187 (40.0 %)	p = 0.484 (C)
Hyperlipidaemia	65 (41.9 %)	68 (42.5 %)	64 (41.8 %)	197 (42.1 %)	p = 0.992 (C)
History of obesity (BMI >30 kg/m^2^)	34 (21.9 %)	32 (20.0 %)	29 (19.0 %)	95 (20.3 %)	p = 0.804 (C)
Symptoms at inclusion					
Typical angina	41 (26.5 %)	40 (25.0 %)	36 (23.5 %)	117 (25.0 %)	p = 0.839 (C)
Atypical angina	63 (40.6 %)	73 (45.6 %)	73 (47.7 %)	209 (44.7 %)	p = 0.439 (C)
Non-angina chest pain	50 (32.3 %)	47 (29.4 %)	44 (28.8 %)	141 (30.1 %)	p = 0.774 (C)
Calcium score	N = 151	N = 152	N = 149	N = 452	
Total score (mean ± SD; range)	154.0 ± 353.4 (0–2288)	204.8 ± 400.9 (0–1972)	135.6 ± 330.5 (0–1890)	165.0 ± 363.4 (0–2288)	p = 0.231(F)
In classes					
Normal (no calcium)	65 (43.0 %)	70 (46.1 %)	72 (48.3 %)	207 (45.8 %)	p = 0.631 (C)
Mild (1–100)	47 (31.1 %)	34 (22.4 %)	45 (30.2 %)	126 (27.9 %)	
Moderate (101–400)	20 (13.2 %)	25 (16.4 %)	15 (10.1 %)	60 (13.3 %)	
High (401–1,000)	11 (7.3 %)	13 (8.6 %)	10 (6.7 %)	34 (7.5 %)	
Severe (>1000)	8 (5.3 %)	10 (6.6 %)	7 (4.7 %)	25 (5.5 %)	
Heart rate	N = 147	N = 156	N = 152	N = 455	
Before CTA (bpm), mean ± SD (range)	61.6 ± 9.0 (45–113)	61.2 ± 9.1 (39–92)	62.0 ± 9.4 (37–90)	61.6 ± 9.2 (37–113)	p = 0.737(F)
During CTA (bpm), mean ± SD (range)	61.5 ± 9.7 (41–103)	61.0 ± 8.9 (40–89)	61.4 ± 10.4 (36–97)	61.3 ± 9.7 (36–103)	p = 0.893(F)
β-blockers	N = 112	N = 111	N = 115	N = 338*	
Intravenous	63 (56.3 %)	60 (54.1 %)	61 (53.0 %)	184 (54.4 %)	p = 0.885 (C)
Oral	49 (43.8 %)	51 (45.9 %)	54 (47.0 %)	154 (45.6 %)	
Radiation dose (post-injection values)	N = 151	N = 152	N = 149	N = 452	
Dose-length product mean ± SD; (range)	415.3 ± 312.3 (38.0–1360.0)	445.9 ± 323.0 (25.0–1562.0)	458.8 ± 307.1 (22.0–1437.0)	439.9 ± 314.1 (22.0–1562.0)	P = 0.469(F)
CT dose index mean ± SD; (range)	26.5 ± 21.5 (0.0–101.0)	28.4 ± 20.7 (1.0–101.0)	30.0 ± 21.4 (1.0–96.0)	28.3 ± 21.2 (0.0–101.0)	P = 0.373(F)

Results are expressed as mean ± standard deviation or n ( %) of patients

* Subgroup of patients who received β-blockers as premedication for CTA

*BMI* body mass index, *CAD* coronary artery disease, *CV* cardiovascular, *CT* computed tomography, *CTA* CTangiography, *F* F-test, *C* Chi-square test

### Image evaluation for detection of stenosis

**Fig. 1:**
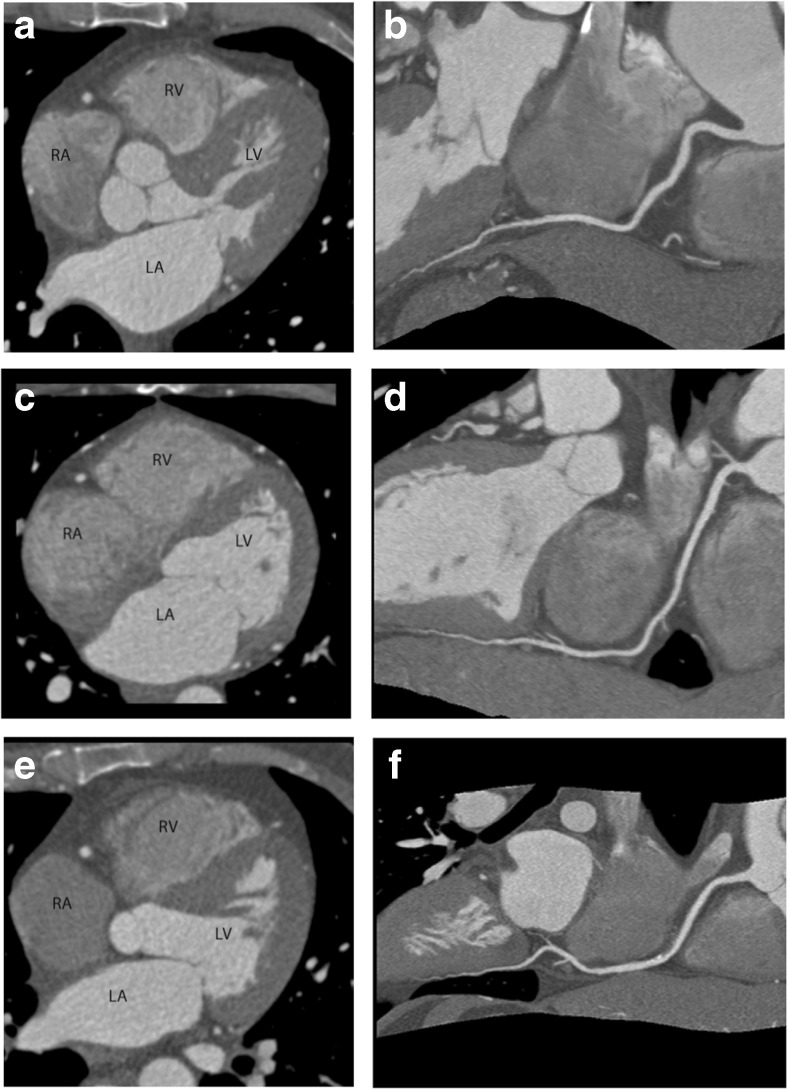
Transaxial cross-sections (0.6-mm slice width) and curved multiplanar reconstructions of the right coronary artery, all displayed at a window level of 1,200 and width of 200 HU. (**a, b**) Investigation performed using iobitridol 350 mg/ml. (**c, d**) Investigation performed using iopromide 370 mg/ml. (**e, f**) Investigation performed using iomeprol 400 mg/ml. *LA* left atrium, *LV* left ventricle, *RA* right atrium, *RV* = right ventricle

The rate of patients with evaluable CT scans was not significantly different between the three groups (92.1 %, 95.4 % and 94.6 % of patients in the FAS, for iobitridol, iopromide and iomeprol, respectively) (Figure 1 and Table 2). The 95 % CI of the difference between iobitridol and the best of the two comparators (iopromide) was [-8.8 to 2.1], demonstrating the non-inferiority of iobitridol, when compared to other CMs, in its ability to allow CAD diagnosis through a complete assessment of coronary artery segments.

**Table 2:** Image quality per patient – off-site evaluation (FAS, N = 452)

	Iobitridol 350	Iopromide 370	Iomeprol 400	All	Test
Patient level	N = 151	N = 152	N = 149	N = 452	
Patients with evaluable CT scans	139 (92.1 %)	145 (95.4 %)	141 (94.6 %)	425 (94.0 %)	p = 0.438 (C)
Number of non–diagnostic segments/patient					
0	139 (92.1 %)	145 (95.4 %)	141 (94.6 %)	425 (94.0 %)	p = 0.181 (C)
1–5	5 (3.3 %)	3 (2.0 %)	1 (0.7 %)	9 (2.0 %)	
6–10	4 (2.6 %)	3 (2.0 %)	4 (2.7 %)	11 (2.4 %)	
>10	3 (2.0 %)	1 (0.7 %)	3 (2.0 %)	7 (1.5 %)	
Average image quality mean ± SD; (range)	3.5 ± 0.9 (0.0–4.0)	3.5 ± 0.8 (0.2–4.0)	3.4 ± 0.9 (0.0–4.0)	3.5 ± 0.9 (0.0–4.0)	p = 0.750 (F)
Segment level	N = 2082	N = 2090	N = 2048	N = 6220	
Image quality					
0 – Non diagnostic	89 (4.3 %)	43 (2.1 %)	71 (3.5 %)	203 (3.3 %)	
1 – Poor quality	65 (3.1 %)	82 (3.9 %)	61 (3.0 %)	208 (3.3 %)	
2 – Moderate quality	143 (6.9 %)	151 (7.2 %)	187 (9.1 %)	481 (7.7 %)	
3 – Good quality	258 (12.4 %)	313 (15.0 %)	334 (16.3 %)	905 (14.5 %)	
4 – Excellent quality	1527 (73.3 %)	1501 (71.8 %)	1395 (68.1 %)	4423 (71.1 %)	

*F* F–test; *C* Chi–square test

The average score for image quality per-segment (total number of segments = 6,220) was 3.5 ± 0.9, 3.5 ± 0.8 and 3.4 ± 0.9 for the iobitridol, iopromide and iomeprol groups, respectively (p > 0.05).

### Attenuation, signal and noise measurements

The average pre-contrast vascular attenuation calculated from values of the ascending aorta, LM and left ventricle was 42.2 ± 9.7 HU, without any difference between the three groups (p = 0.993; Table 3). Vascular attenuation was significantly increased in post-contrast images as compared to pre-contrast images in all three structures. Average post-contrast arterial vascular attenuation was 426.3 ± 92.9 HU, 449.8 ± 88.1 HU and 466.4 ± 104.6 HU for the iobitridol, iopromide and iomeprol groups, respectively (p = 0.001).The difference between groups was statistically significant for absolute values; however, when values accounting for noise were plotted as SNR and CNR, differences were no longer significant (Table 3). Measurements of noise in the ascending aorta showed no significant difference between groups (p = 0.311).

**Table 3:** Signal quantification at patient level - off-site evaluation (FAS, N = 452)

	Iobitridol 350	Iopromide 370	Iomeprol 400	All	p-value (F-Test)
	N = 151	N = 152	N = 149	N = 452	
Attenuation					
Ascending aorta					
Pre	44.8 ± 9.9	44.2 ± 10.3	44.7 ± 11.3	44.6 ± 10.5	0.864
Post	440.0 ± 94.0	465.0 ± 93.6	476.8 ± 114.9	460.6 ± 102.2	0.006
Left ventricle					
Pre	42.0 ± 11.6	41.1 ± 10.7	41.4 ± 11.1	41.5 ± 11.1	0.789
Post	382.3 ± 108.8	401.2 ± 113.7	431.1 ± 124.3	404.7 ± 117.2	0.001
Left main coronary artery					
Pre	39.7 ± 14.5	41.1 ± 15.7	40.8 ± 13.4	40.5 ± 14.6	0.666
Post	441.9 ± 101.0	466.8 ± 100.8	479.9 ± 116.6	462.8 ± 107.2	0.008
Average pre-contrast	42.2 ± 9.7 (21.1–90.5)	42.2 ± 10.2 (15.3–92.0)	42.3 ± 9.4 (19.9–84.6)	42.2 ± 9.8 (15.3–92.0)	0.993
Average post-contrast	426.3 ± 92.9 (240.6–726.2)	449.8 ± 88.1 (240.3–717.4)	466.4 ± 104.6 (185.5–766.9)	447.4 ± 96.6 (185.5–766.9)	0.001
Average noise aorta	32.5 ± 10.5 (15.5–80.5)	32.8 ± 10.0 (13.8–69.2)	34.3 ± 11.5 (14.3–75.7)	33.2 ± 10.7 (13.8–80.5)	0.311
Average SNR	16.2 ± 5.6 [7.0–45.7)	17.1 ± 5.0 (6.5–31.4)	17.6 ± 6.6 (5.2–46.4)	17.0 ± 5.8 (5.2–46.4)	0.109
Average CNR	14.4 ± 5.4 (5.6–42.8)	15.3 ± 4.8 (5.8–28.7)	15.8 ± 6.5 (4.3–48.0)	15.2 ± 5.6 (4.3–48.0)	0.090

All values are expressed as Hounsfield Units (HU) with mean and standard deviation. The range is provided in brackets for average values.

*FAS* full analysis set, *CNR* contrast to noise ratio, *SNR* signal to noise ratio, *Pre* prior to contrast media intravenous administration, *Post* after contrast media intravenous administration, *SD* standard deviation

### Other secondary endpoints

No difference was observed regarding the number of significant stenoses identified with the three CMs (p = 0.580; Table 4).

**Table 4:** Secondary efficacy endpoints

Secondary endpoints	Iobitridol 350	Iopromide 370	Iomeprol 400	Total	Test
Pain assessment	N =148	N =148	N =144	N = 440	
Pain during and after examination - VAS (cm)	0.4 ± 0.9	0.5 ± 1.0	0.7 ± 1.5	0.6 ± 1.2	p = 0.049 (F)
Patient comfort	N = 150	N = 151	N = 148	N = 449	
Comfort score*	4.4 ± 0.6	4.4 ± 0.6	4.3 ± 0.6	4.4 ± 0.6	p = 0.347 (F)
Patient management	N = 151	N = 152	N = 149	N = 452	
No action	107 (70.9 %)	109 (71.7 %)	114 (76.5 %)	330 (73.0 %)	p = 0.494 (C)
Medication	22 (14.6 %)	24 (15.8 %)	19 (12.8 %)	65 (14.4 %)	p = 0.752 (C)
Invasive coronary angiography	20 (13.2 %)	18 (11.8 %)	14 (9.4 %)	52 (11.5 %)	p = 0.572 (C)
Other recommendation	6 (4.0 %)	6 (3.9 %)	8 (5.4 %)	20 (4.4 %)	p = 0.791 (C)
Stenosis (>50 %) assessment					
Per-segment	N = 2718	N = 2736	N = 2682	N = 8136	p = 0.003 (C)
Certainly no	1670 (84.3 %)	1729 (83.4 %)	1753 (86.5 %)	5152 (84.8 %)	
Probably no	235 (11.9 %)	263 (12.7 %)	211 (10.4 %)	709 (11.7 %)	
Doubtful	26 (1.3 %)	32 (1.5 %)	26 (1.3 %)	84 (1.4 %)	
Probably yes	29 (1.5 %)	17 (0.8 %)	28 (1.4 %)	74 (1.2 %)	
Certainly yes	20 (1.0 %)	31 (1.5 %)	8 (0.4 %)	59 (1.0 %)	
Per patient	N =151	N = 152	N = 149	N = 452	
overall significant stenosis (doubtful to certainly)	31 (20.5 %)	30 (19.7 %)	24 (16.1 %)	85 (18.8 %)	p = 0.580 (C)
Coronary track rate	N = 151	N = 152	N = 149	N = 452	
number of tracked segments per patient	10.9 ± 2.2	10.8 ± 2.4	11.1 ± 2.3	10.9 ± 2.3	

Results are expressed as mean ± standard deviation or n (%) of patients or segments

*VAS* visual analogue scale (10 cm), *F* F–test; *C* Chi–square test

*Comfort during examination was rated by the patient on a scale from 1 (very poor) to 5 (very good)

Multivariate analyses showed a relationship between calcium scoring and stenosis assessment according to territory (p < 0.001) and between calcium scoring and image quality regardless of the territory (p = 0.007).

A mean of 11 segments were automatically tracked out of a maximum of 13 segments per patient whatever the contrast media injected (Table 4).

The mean score for comfort of the examination rated by the patient was good (4.4 ± 0.6) and similar for all three groups. Patient comfort was confirmed by a low reported intensity of pain (mean score of less than 1 out of 10 cm on VAS for the three groups).

Regarding patient management, no action was required after the CTA for 73 % of the patients overall, with no significant difference between groups.

### Clinical safety

The percentage of patients experiencing post CM-injection AEs was 15.1 %; 19.5 % and 15.1 %, for the iobitridol, iopromide and iomeprol groups, respectively (Table 5). Most AEs concerned cardiac disorders, which were reported through systematic ECG follow-up performed up to 10 min post-injection. Overall, mean heart rate was 65.0 ± 9.7 bpm 2 min after CM injection and 62.8 ± 9.3 bpm 10 min after CM injection, and was similar in all three groups.

**Table 5:** Incidence and characteristics of adverse events (safety set, N = 463)

Adverse events	Iobitridol 350	Iopromide 370	Iomeprol 400	All
	N = 152	N = 159	N = 152	N = 463
n ( %) of patients with at least one:				
Pre CM-injection AE	24 (15.8 %)	14 (8.8 %)	15 (9.9 %)	53 (11.4 %)
Post CM-injection AE	23 (15.1 %)	31 (19.5 %)	23 (15.1 %)	77 (16.6 %)
Number of post CM-injection AE	26	36	28	90
Intensity				
Mild	26 (100.0 %)	32 (88.9 %)	20 (71.4 %)	78 (86.7 %)
Moderate	0 (0.0 %)	4 (11.1 %)	7 (25.0 %)	11 (12.2 %)
Severe	0 (0.0 %)	0 (0.0 %)	1 (3.6 %)	1 (1.1 %)
Outcome				
Resolved	26 (100.0 %)	34 (94.4 %)	27 (96.4 %)	87 (96.7 %)
Ongoing	0 (0.0 %)	2 (5.6 %)	1 (3.6 %)	3 (3.3 %)
Relationship to CM				
Not related	12 (46.2 %)	22 (61.1 %)	18 (64.3 %)	52 (57.8 %)
Doubtfully related	12 (46.2 %)	9 (25.0 %)	5 (17.9 %)	26 (28.9 %)
Possibly related	2 (7.7 %)	5 (13.9 %)	5 (17.9 %)	12 (13.3 %)

*AE* adverse events, *CM* contrast media

One patient could have experienced several adverse events

No severe AEs were reported. Only mild events were reported with iobitridol while four and seven moderate events were reported with iopromide and iomeprol, respectively. Few post-CM AEs were considered possibly related to CM administration: two in the iobitridol group and five in the iopromide group as well as in the iomeprol group. The cardiac events considered possibly related to CM injection were bradycardia (one patient in each group) and extrasystoles (two patients in the iomeprol group). Other possibly related events were pain in the iobitridol group, injection site pain, nausea, headache and urticaria in the iopromide group, injection site pain and feeling hot in the iomeprol group.

## Discussion

The rationale for this trial was that CM iodine concentration may not play a role in the ability to visualize coronary stenoses by CTA. This study demonstrated the non-inferiority of iobitridol 350 in providing evaluable CT scans for assessment of coronary stenosis, as compared to CM with higher iodine concentrations (iopromide 370 mg iodine/ml and iomeprol 400 mg iodine/ml). These two CMs had been compared in a previous study without differences in terms of image quality [25]. Our study, the largest so far to address the relationship between iodine concentration and image quality, indicates the possibility of further reducing iodine content. Reflecting current clinical practice in many sites, IDR was not adjusted according to the iodine concentration of each CM.

Our results are in line with previous studies in which higher iodine concentrations were associated with higher intravascular attenuation [14, 31–34]. However, comparison is difficult since the volume of iodine injected was not always adjusted to patient weight [14]. The fact that no difference was observed between CMs when considering SNR and CNR cannot be fully explained. Compared to previous studies, image noise was relatively low given the advanced technology of the CT systems that were used. This limits the influence of noise. Also, statistically, variations in image noise across patients and systems may have been larger than variations in contrast enhancement, so that lack of significance regarding the difference between the three patient groups may be a statistical effect.

Recent improvements in terms of radiation dose management also affect vascular attenuation and noise. For instance, lower kV protocols (80 kV) can now be used for patients with small body mass indices (BMIs) allowing for a reduction in radiation dose while increasing vascular attenuation [6]. In this study, no protocol using less than 100 kV was performed. Another recent development affecting noise is related to the introduction of iterative reconstruction algorithms which are able to significantly reduce image noise thus improving SNR and CNR while keeping intravascular attenuation constant [6, 9]. It is therefore reasonable to expect that using lower kV settings and an iterative reconstruction algorithm may have yielded better SNR values regardless of other parameters. However, the inclusion of iterative reconstruction algorithms might have hampered comparisons to images from other scanners unless image noise would have been adapted by lowering the radiation exposure during the acquisition. Finally, newer detectors have been introduced with increased efficiency and lower noise [5] hence promoting the use of less iodine content (less volume or lower concentrations [35–40]).

Regarding safety, iobitridol use was associated with the lowest incidence of related AEs (i.e. 1.3 % corresponding to two patients), confirming the excellent safety profile of this CM [41–43].

### Study limitations

The main limitation of the study is the absence of a gold standard such as conventional angiography. Diagnostic accuracy could therefore not be compared between groups. A similar number of scans positive for stenosis in all three groups, however, indicate that there is likely no systematic difference in stenosis detection rates between the three CMs.

Another limitation is the relatively low coronary calcium burden. This is typical for the target population of the study. While severely calcified vessels may benefit from increased vessel lumen opacification for reliable evaluation [16, 17], this was not specifically evaluated and the ability to identify calcified lesions was not compared across the three CMs. A systematic assessment of renal function post-contrast was not performed. Further, contrast injection rates for the three CMs used were constant and not adjusted to achieve equal IDR across the three groups. Hence IDR ranged from 1,400 to 1,600 mg iodine/s in the patients with the lowest body weight and from 2,100 to 2,400 mg iodine/s in patients with the highest body weight. This reflects clinical practice, where protocols typically prescribe injection rates in ml/s and not IDR, and explains why higher attenuation values were observed for higher concentration CMs, but did not lead to significant differences regarding SNR, CNR or the clinical rating of image quality.

## Conclusion

With current CT technology, iobitridol 350 mg iodine/ml is not inferior to CMs with higher iodine in terms of image quality for coronary stenosis assessment by CTA. When considering image quality, SNR and CNR, iobitridol yielded similar values to iopromide and iomeprol. Iobitridol, with a lower content of iodine, holds the potential to reduce the risk of adverse reactions, as supported by its excellent safety profile. It is likely that developments in image reconstruction and detector technology may further allow improving image quality while minimizing the necessary amount of injected iodine.
